# Drug delivery for neurodegenerative diseases is a problem, but lipid nanocarriers could provide the answer

**DOI:** 10.7150/ntno.88849

**Published:** 2024-01-01

**Authors:** Md. Rajdoula Rafe

**Affiliations:** 1 Department of Neuroscience, City University of Hong Kong, Kowloon, Hong Kong SAR, China.; 2 Department of Pharmacy, Jagannath University, Dhaka-1100, Bangladesh.

**Keywords:** nanoparticles, neurodegenerative disorders, blood-brain barrier, nanocarriers, central nervous system (CNS)

## Abstract

Neurodegenerative disorders encompass diseases that involve the degeneration of neurons, particularly those within the central nervous system. These are the most commonly observed disorders among the geriatric population. The treatment or management of this condition presents additional challenges due to therapeutics that may not be as effective as desired. The primary obstacle that often hinders the efficacy of therapy is the existence of a blood-brain barrier (BBB). The BBB serves as a vital safeguard for the brain, effectively obstructing the passage of drugs into the brain cells. Hence, the management of damaging neurodegenerative conditions such as Alzheimer's disease (AD), Parkinson's disease (PD), Cerebrovascular diseases (CVDs), Huntington's disease (HD), and Multiple sclerosis (MS) is currently the primary area of research interest. The innovative utilization of nanoparticles as drug carriers provides renewed optimism in addressing many complicated medical conditions. In this article, I have aimed to gather published information regarding various lipid nanoparticles that can efficiently transport medication to the brain to address neurodegenerative disorders. According to the published literature, liposomes, solid-lipid nanoparticles, nanostructured nanoparticles, microemulsions, and nanoemulsions are potential nanocarriers that can treat neurodegenerative disorders.

## 1. Introduction

To function and transmit electrical signals efficiently, the neurons of the CNS need a constant supply of nutrients and other gases 1. To maintain a perfect brain environment, the fluids surrounding neurons and shielding them from mechanical disturbances must be tightly regulated 2. Brain cells are not only protected by fluids but also by the blood-brain barrier (BBB). The physical barrier known as the BBB comprises specialized endothelial cells, astrocytes, pericytes, and neurons that keep the brain in a state of homeostasis by closely maintaining the flow of chemicals into the CNS 3.

The generation of neurodegenerative disorders such as Alzheimer's disease (AD), Parkinson's disease (PD), Huntington's disease (HD), Multiple sclerosis (MS), or Amyotrophic lateral sclerosis is facilitated by pathogenic processes that affect the CNS 4. To protect the brain from toxic and poisonous substances blood-brain barrier is necessary, but it also serves as a barrier to drug entry due to its higher selectivity 4. The discovery of novel medications for CNS disorders, the BBB, poses a significant limitation. Certain chemicals cannot pass the barrier to enter the brain. Peptides, recombinant proteins, monoclonal antibodies, RNA interference (RNAi)-based medications, and gene therapy drugs are large molecules that usually do not penetrate the BBB 5.

The literature reports several methods to administer medications to the brain, including intranasal administration and intracerebroventricular and intracerebral administrations, to pass the BBB in response to different stimuli 6. High drug loading, improved physical and chemical stability, and low toxicity of carrier molecules should all be considered when building a drug delivery system 7. The main problem is creating a model that maintains the BBB's basic properties while remaining compatible with the drug's effectiveness 8. The most effective way to get the right medications into the CNS is still unclear for many disorders 9. Various nanocarriers have been produced and used as CNS delivery systems for diagnostic and therapeutic reasons, enhancing biological distribution and pharmacokinetics and raising drug concentration in the brain 10. Due to their unique optical, thermal, magnetic, and physicochemical characteristics, such as small size, large surface area, strong macromolecule adsorption capacity, and high chemical reactivity, materials with mean sizes ranging from 0.1 nm to 100 nm aim to cross the BBB have drawn attention 11, 12.

Nanomedicines are expected to help drugs pass the BBB, boosting their bioavailability based on their superior qualities 12. However, polymeric nanoparticles are not as effective as lipid nanocarriers. The utilization of lipid nanoparticles presents a promising avenue for overcoming the obstacles commonly encountered with polymeric nanoparticles, including the undesirable cytotoxic effects and the absence of viable approaches for efficient large-scale manufacturing 13, 14. Lipid-based novel drug delivery systems have been primarily centered around transporting lipophilic molecules. Nonetheless, there has been a recent surge of interest in lipoid drug delivery systems, primarily due to their fundamental characteristics such as biocompatibility, self-assembly capabilities, capacity to traverse the BBB, variability in particle size, and cost-effectiveness. These qualities render lipid-based delivery systems considerably more appealing 15, 16. Due to their efficiency in encapsulating and crossing biological membranes to carry lipophilic drugs, lipid nanoparticles, such as solid lipid nanoparticles (SLNs), liposomes, nanoemulsion, and nanostructured lipid carriers (NLCs), are the most promising drug delivery systems that have been suggested by researches 17-19. Specifically, SLNs have gained attention as a potential drug delivery system that has the potential to deliver drugs to the specific target site of the brain after passing BBB effectively. This new strategy offers controlled drug delivery, a longer circulation time, higher target specificity, and efficacy 20.

This review aimed to incorporate research advances in developing lipid-based nanocarrier therapeutics for their implications in treating neurodegenerative disorders.

## 2. Drug Administration Complexities Due to BBB

**Figure 1:**
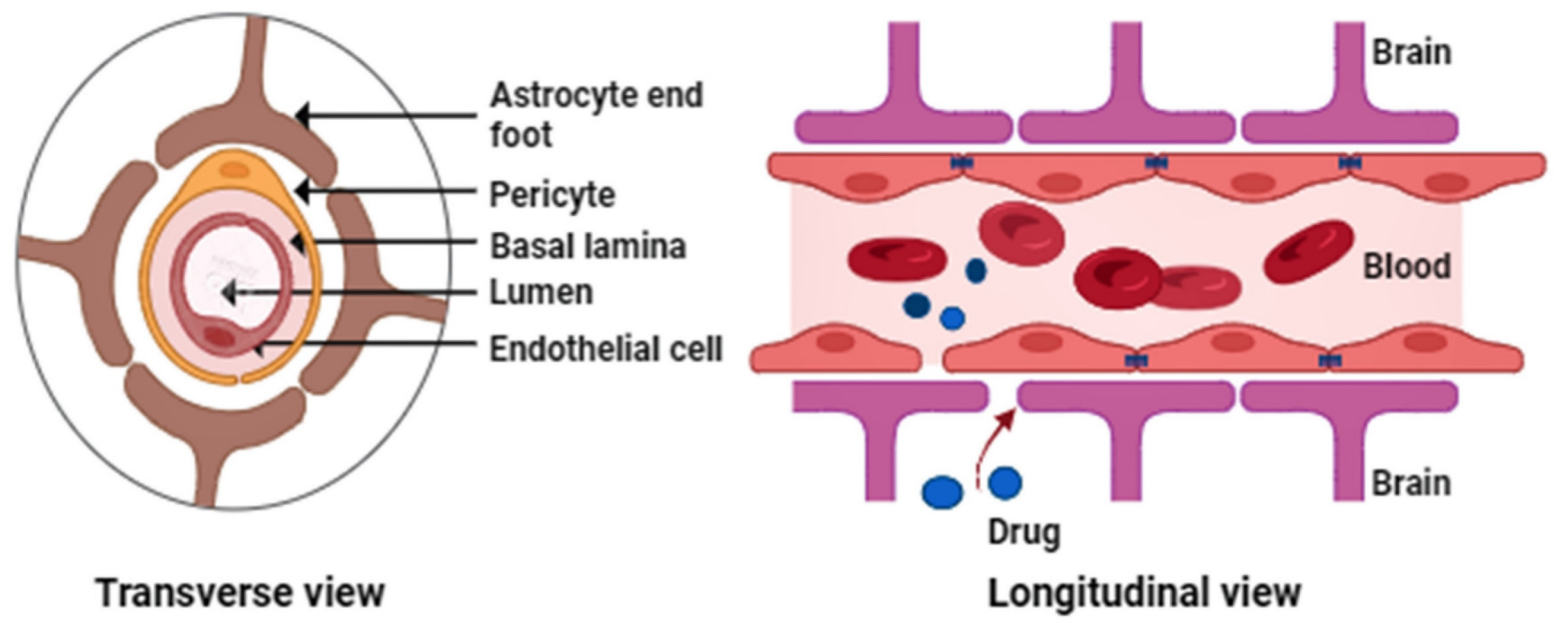
Generalized figure of the blood-brain barrier physiology.

The BBB is essential to the body's neurovascular system, communicating with the CNS. It is well known that the BBB is the primary impediment to properly treating neurological disorders because it limits the CNS from receiving a variety of potentially helpful therapeutic and diagnostic substances. It also limits the free flow of chemicals between brain cells 21. The major BBB constituents (Figure 1) include endothelial cells, astrocytic end-foot connections, basal lamina, tight junctions, and pericytes 22-23.

Preventing toxic substances from entering the brain is possible by maintaining peripheral circulation from the CNS 25. Through passive transport, the BBB performs filtering tasks and selectively permits the passage of substances, including water, nutrients, and hydrophobic compounds 26. The blood-cerebrospinal fluid barrier (BCSFB) adds another barrier to treatments and increases CNS complexity. The choroid plexus is a collection of ependymal cells that also serve as a barrier in the brain by isolating blood from cerebrospinal fluid (CSF) 27. The BBB and other efflux transporters are crucial in ineffective drug absorption into the brain. The BBB has many ATP-dependent efflux transporters 25. BBB's active efflux systems and overexpressed tight junctions also prevent medicines from reaching their therapeutic target. P-glycoprotein (P-gp), an ABC transporter, efficiently removes drugs from the brain and pumps them back into circulation 28.

## 3. Neurodegenerative Disorders

**Figure 2:**
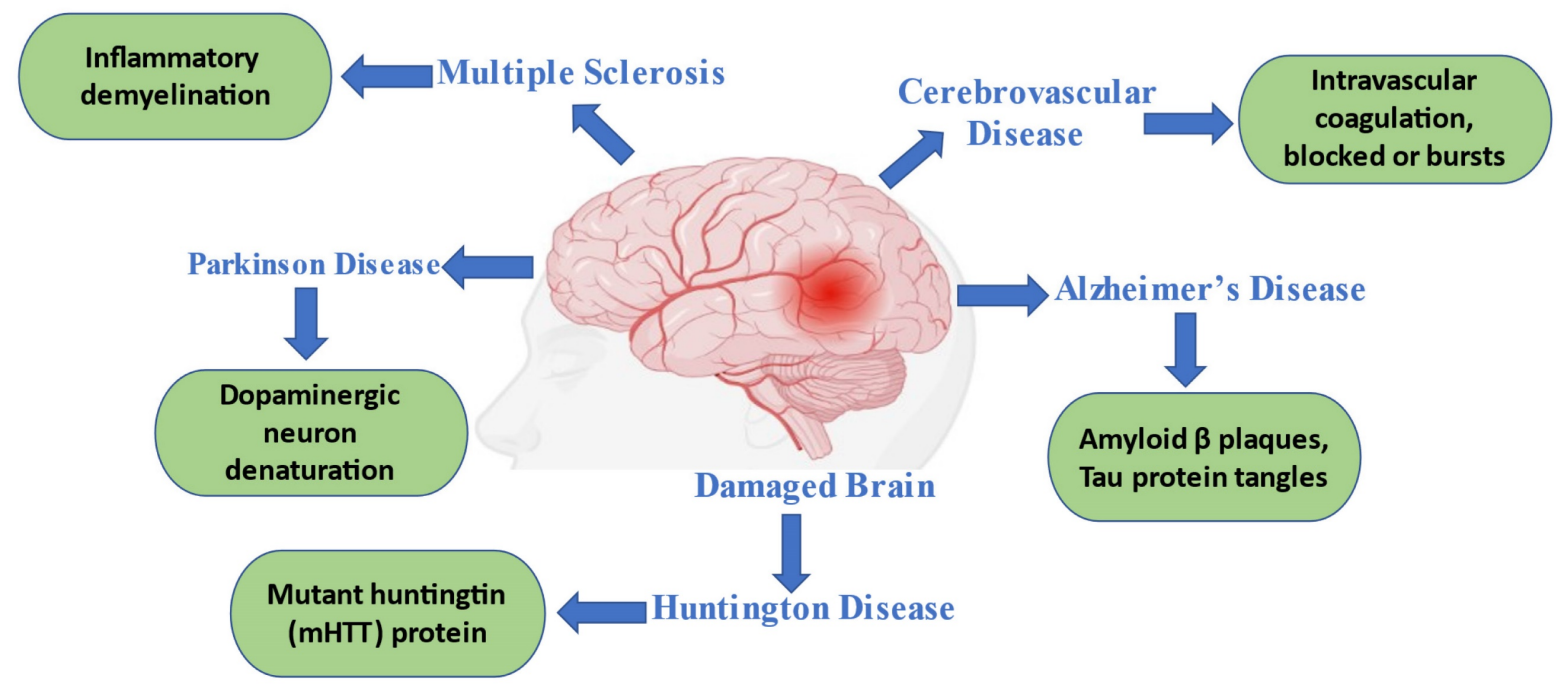
Common neurodegenerative disorders with their pathophysiological indications.

Age-related neurodegenerative diseases (NDs) are widespread. Although the peripheral neurological system (PNS) is also affected, the CNS is affected the most among the aging population 29. Neuronal brain and spinal cord loss are a hallmark of many diseases. Alzheimer's, Parkinson's, Huntington's illnesses, multiple sclerosis, and amyotrophic lateral sclerosis are a few examples of neurodegenerative diseases (Figure 2). Clinical characteristics of each disease vary depending on the CNS regions implicated; some, for example, cause cognitive and memory problems, while others impair speech, locomotion, and breathing abilities. The development of novel and more potent treatments, which are desperately needed, can only be sped up by a greater understanding of the pathophysiology and pathogenesis of diseases 9, 30.

### 3.1 Alzheimer's Disease (AD)

The primary cause of dementia in late adulthood is Alzheimer's disease, which is recognized as a progressive, multifaceted neurological condition 31. Alois Alzheimer first introduced the concept of AD in 1906. Amyloid- β is deposited extracellularly as plaques, hyperphosphorylated tau protein aggregates intracellularly as tangles, and intra-cortical projecting neurons gradually degenerate in AD 32. Acetylcholinesterase inhibitors and N-methyl-D-aspartate receptor antagonists are two therapy options 33. New tactics have been devised to alter the illness process. Aβ and tau-based therapies are currently the focus of significant research and development in this area, and they hold the key to curing this disease in the near future 34, 35.

### 3.2 Parkinson's Disease (PD)

Dopaminergic neuron denaturation presence in substantia nigra causes striatal dopamine reduction in PD, the second most prevalent neurodegenerative illness 36, 37. The condition progresses with a gradual loss of motor control, which causes severe respiratory and gastrointestinal issues that ultimately result in the patient's death 38. Although the cornerstone of PD treatment is dopamine supplementation, the acetylcholine, norepinephrine, and serotonin systems are also found to be defective in Parkinson's disease 39-43. Effective preliminary treatments include levodopa preparations, dopamine agonists, and monoamine oxidase-B (MAO-B) inhibitors. Anticholinergic medications, such as trihexyphenidyl, are helpful for young people with severe tremors. Still, they should be used with caution due to the possibility of side effects, particularly those that could affect cognition 44.

### 3.3 Cerebrovascular Diseases (CVDs)

All illnesses that predominantly affect the brain's blood arteries are called cerebrovascular disorders 45. The most frequent symptom of cerebrovascular condition is stroke. It happens when a cerebral artery becomes blocked or bursts 46. There are two major types of strokes: ischemic stroke, which involves occlusions of cerebral vessels, and hemorrhagic stroke, which involves intracerebral hemorrhage (ICH) 47. Hemorrhagic stroke can be brought on by chemotherapeutic side effects, cancer-related coagulopathies, particularly those from leukemia, and metastatic brain illness. Additionally, ischemic stroke can result from chemotherapy, non-bacterial thrombotic endocarditis (NBTE), a pro-thrombotic condition such as disseminated intravascular coagulation (DIC), or metastatic illness with local vessel invasion 48.

### 3.4 Multiple Sclerosis (MS)

MS usually affects young people and causes non-traumatic debilitating conditions 49. MS develops gradually, affecting the spinal cord, brain stem, basal ganglia, visual neurons, and other CNS white matter 50. An individual's genetic makeup, Epstein-Barr virus, sunlight, smoking, and vitamin D play significant roles in MS development 51. Disease-modifying medicines and symptomatic therapy are both used to treat the symptoms of MS, which are brought on by neurological problems 49. Interferons, glatiramer acetate, teriflunomide, sphingosine 1-phosphate receptor modulators, fumarates, cladribine, and three different kinds of monoclonal antibodies are commonly used for the treatment of MS 52.

### 3.5 Huntington's Disease (HD)

Dementia, behavioral and psychological issues, and uncontrollable choreatic movements indicate Huntington's disease (HD), a relatively uncommon neurodegenerative disease 53, 54. George Huntington originally described HD, also known as hereditary chorea, in 1872. He talked about the genetic origin of the condition, how the disease occurs in individuals between the ages of 30 and 40, and psychological and cognitive symptoms 55. Chromosome 4 has the huntingtin (HTT) gene, which increases the CAG trinucleotide repeats and causes HD. It causes the development of a mutant huntingtin (mHTT) protein having an extensive polyglutamine repeat 56. The symptoms they address can help to categorize the current HD treatments. These categories include non-drug therapy, antipsychotic drugs, antidepressants, mood stabilizers, and chorea medications 57.

## 4. Applications of Nanoparticles (NPs) to Cross the BBB

The chemistry, design, and characteristics of the NPs dictate the possible method of NP-mediated drug transport through the BBB 58. Since they have a small size and high surface-to-volume ratio, NPs make excellent candidates for use as drug carriers. The medications they carry can be released more quickly and have higher bioavailability because they are closer to the surface 59. To deliver loaded pharmaceuticals to the desired site of action with improved release kinetics and increased therapeutic efficacy with maximum therapeutic benefits, precisely modified nanocarriers are required 60-62.

**Figure 3:**
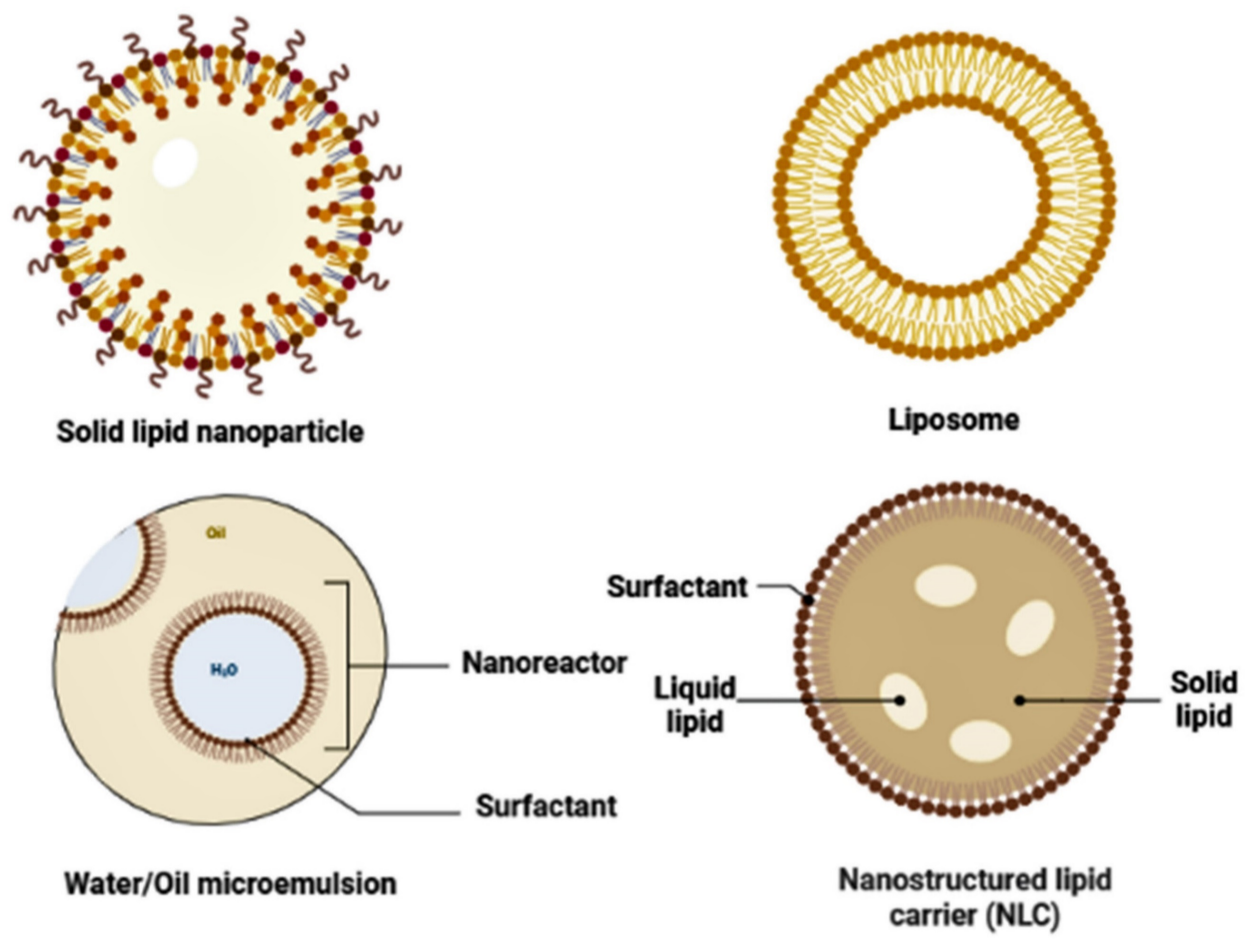
Different types of lipid nanoparticles used for the effective delivery of drugs.

It should be emphasized that non-modified NPs administered systemically frequently interact with serum proteins unintendedly. In the reticuloendothelial system (RES), which is present mainly in the liver and spleen, opsonin adsorption on the surface creates a macrostructure known as a "corona," and this corona reported to be easily absorbed and destroyed by macrophages 63. Coating NPs with hydrophilic polymers or surfactants is a commonly used method for resolving this problem; amphipathic polyethylene glycols have received much interest in investigating this topic 64, 65. Nanocarriers can be made from various substances, such as carbon nanotubes, liposomes, micelles, polymeric and lipid-based nanoparticles (Figure 3), dendrimers, and micelles 66.

## 5. Lipid-based Nanocarriers

### 5.1 Solid Lipid Nanocarriers (SLNs)

Phospholipids are a crucial part of lipid and lipid-based drug delivery systems due to their many properties, including amphiphilic behavior, biocompatibility, and multi-functionality. The multiple drawbacks of liposomes, lipospheres, and microemulsion carrier systems, such as their difficult large-scale fabrication, poor percentage entrapment efficiency (% EE), and hard production process, have led to the development of the SLN delivery system 67-69. When making SLN, solid lipids at body and room temperatures are often used, and they are stabilized using one or more surfactants 70. The lipids used can be waxes, combinations of complicated glycerides, or highly purified triglycerides 70.

### 5.2 Liposome

In intranasal delivery, particularly for neurological diseases, liposomes-artificial lipid-based bilayered vesicles have become one of the most effective and popular lipid nanocarriers 71. Typically, liposomal preparation comprises dissolving cholesterol, lecithin, and occasionally charge-inducing agent(s) in an organic solvent before drying to create a thin film. A liposome suspension is then made by dispersing the film in an aqueous solution at a crucial hydration temperature 72. According to lipophilic properties, liposomal nanocarriers' most significant characteristic is protection from degradation and optimization of the pharmacokinetics of the encapsulated drug. These nanocarriers also release their content after phagocytosis and nonphagocytic endocytosis, which enables encapsulated drugs to enter the brain 73.

### 5.3 Nanostructured Lipid Nanocarriers (NLCs)

Similar to SLN in terms of physicochemical characteristics, NLC was created by nanostructuring the architecture of the lipid matrix to increase API loading while also preventing drug leakage during storage, giving them more flexibility to target particular API release profiles 74,75. A second generation of lipid nanoparticles called NLCs was created to enhance drug loading 76. NLCs are binary systems that contain solid and liquid lipids, which leads to a less organized lipidic core 77. Although both SLNs and NLCs are useful for increasing the solubility of drugs in aqueous solutions and aiming for targeted drug release in the brain to treat various neurological disorders, the production process of NLCs and SLNs is the same. However, there are notable differences between the two dosage forms that only affect overall performances and the fate of such systems due to the types (nature) of lipids used to form their Matrigel 78.

### 5.4 Microemulsions (MEs) and Nanoemulsions (NEs)

An optically isotropic, thermodynamically stable system with two phases—an aqueous phase and a lipid phase- is called a microemulsion (ME) 79, 80. The biphasic dispersion of two incompatible liquids, either oil in water (O/W) or water in oil (W/O) droplets stabilized by an amphiphilic surfactant, is what constitutes nanoemulsion (NE) 77. There are some differences between the two sorts of systems. NEs are non-equilibrium, thermodynamically unstable systems as opposed to MEs 82, 83. To increase stability, delivery, and bioactivity, numerous synthetic and natural chemicals have been produced. Nanoemulsions and microemulsions offer a good choice for the administration of drugs through lipophilic barriers 84.

## 6. Treatment of Common Neurodegenerative Diseases with Lipid Nanocarriers (Table 1)

### 6.1 Alzheimer's Disease

According to Dhawan et al., tween 80 and compritol were used to make SLN, which then encapsulated quercetin. This SLN-encapsulated quercetin gives good results in the aluminum-induced rats model to improve memory retention 85. Quercetin-loaded nanosystems prepared by Pinheiro et al. show promise for treating neurological illnesses like AD, especially NLC, due to their improved capacity to transport quercetin to particular brain locations as well as their improved ability to inhibit amyloid- β aggregation 86.

RVG29-nanoparticles, by Pinheiro et al., target the BBB and stimulate neuronal defense against amyloid-beta fibrillation 87. It is a potentially effective way to distribute quercetin and a strategy that holds promise for Alzheimer's disease treatments in the future 87. Findings from a study suggested that innovative EPO (erythropoietin)-loaded solid lipid nanoparticles may hold promise for treating neurodegenerative illnesses since they efficiently sustain memory in an animal model of AD 88.

A study's findings support a neuroinflammation model that mimics AD, in which inflammatory cells promote the synthesis of cytokines that cause inflammation while affecting learning and spatial memory. Intriguingly, MET-PSL (Metformin-loaded phosphatidylserine nanoliposomes) formulation may be more effective in treating AD in rats. It may suppress neuroinflammation markers like IL-1, TNF, and TGF-cytokines, improve neural tissue and cells, and restore learning and memory. The MET-PSL formulation may help AD patients with learning and memory problems and manage unchecked neuroinflammation 89.

Additionally, Jiang et al. showed intranasal transport of a Huperzine A-loaded lactoferrin-linked nanoemulsion to the brain. The hypothesized drug transport mechanism, receptor-mediated transcytosis, demonstrated the effectiveness of lactoferrin for brain targeting. Better pharmacokinetic and animal study data added credence to the idea that intranasal nanoemulsion would be helpful in AD site targeting 90.

### 6.2 Parkinson's Disease

In a study, hydrogel formulations containing RP (ropinirole) loaded lipid nanoparticles were successfully created and improved. Studies verified the RP's sustained and protracted release and its improved permeability. The lipid nanoparticles and hydrogel formulations showed enhanced oral and transdermal bioavailability upon PK studies. PCD studies demonstrate the repair of metabolic alterations in the rat PD model. The outcomes showed that SLN and NLC-enhanced hydrogel formulations could be considered alternative administration techniques to deliver RP for parkinsonism 91.

Uppuluri et al. designed and refined Piribedil (PBD) loaded SLNs. To facilitate effective intravenously administered drug administration, the optimized PBD-SLNs (Piribedil-loaded SLNs) were also loaded in Methyl Cellulose (MC)-based thermo-responsive in situ gels (PBD-SLN-ISG). Compared to intravenously administering a simple PBD suspension (PBD-Susp), the developed PBD-SLN-ISG demonstrated considerable nose-to-brain delivery with a 4-fold higher brain availability of PBD. These findings demonstrated the superiority of the created PBD-SLNs over the currently used traditional oral PBD therapy for treating Parkinson's disease 92.

In vivo, testing of *Bacopa monnieri* neuroprotective effects on rotenone-induced Parkinson's disease rats revealed that *B. monnieri*-SLNs loaded microneedle patches had superior neuroprotective action than pure drug 89. The ROT (rotenone)-induced PD rodent model was used to assess the neuroprotective activity of naringenin-SLN in research by Mani et al. The findings of behavioral studies and biomarkers shed light on the idea that naringenin, an SLN, may have neuroprotective effects and potentially slow the course of PD 94. Rahman et al. demonstrated that solid lipid nanoparticles with vitexin were effective against 6-hydroxydopamine-induced neurotoxicity in rats. It could be an additional, successful therapeutic strategy for managing PD 95.

### 6.3 Cerebrovascular Diseases

An experiment on brain distribution revealed that NLCs considerably improved the brain-targeting effectiveness of baicalein. In the central brain, NLCs effectively attack the cortex and brain stem. Vitamin E and gelucires may significantly impact pharmacokinetics and brain transport. It could be an efficient drug-targeting method for treating CNS illnesses and brain traumas because Tocol NLCs increase baicalein's stability and capacity to enter the brain 96. In the stroke model, Ferulic acid (FA)-NLCs offer longer-lasting therapeutic effects by enhancing the pharmacological profile of FA. This nanoformulation may be a suitable controlled-release drug carrier system against ischemia injuries or other neurodegenerative disorders 97.

Gao et al. showed that compared to intravenously or orally administered daidzein suspension, the pharmacokinetic behavior of SLNs loading daidzein demonstrated that it may considerably lengthen circulation duration. Compared to oral suspension or intravenous solution, SLNs had a superior effect on the cardiovascular system of the anesthetized dogs. The SLNs outperformed the other two formulations regarding their ability to improve cerebral blood flow (CeBF) and lower cerebrovascular resistance (CeR) in sedated dogs and their ability to protect rats using an ischemia-reperfusion damage paradigm. These results suggest that SLNs loaded drugs may be a candidate for the therapy of cardio-cerebrovascular diseases. 98.

According to studies, the preferential accumulation of liposomes inside the lesion site is related to a biphasic pattern of BBB hyper-permeability. It offers a rare chance to transfer therapeutic molecules over the BBB selectively and effectively, a method that can be used for hemorrhagic stroke therapy 99. All the symptomatic parameters of strokes of the MCAO (middle cerebral artery occlusion) rats were significantly improved by oleoylethanolamide (OEA) loaded LNPs, according to in vivo investigations. These findings strongly suggest that the produced nanoparticles will transform hydrophobic OEA into a possible therapy for stroke 100. Sabry et al. claimed that a promising method for reducing the detrimental effects of stroke is the delivery of Valsartan to the brain using SLNs tagged with Rhodamine B 101.

### 6.4 Multiple Sclerosis

The findings of a study showed that DMF (Dimethyl Fumarate) loaded controlled release SLNs could be a promising choice for the enhancement of multiple sclerosis disease management 102. Kumar et al. targeted treating neurological disorders like multiple sclerosis by using SLNs to orally transport methylthioadenosine (MTA) to the brain. The SLNs of stearic acid were smaller than 100 nm and provided more drug loading and entrapment. The pharmacokinetic studies' evidence for better bioavailability supported the pharmacodynamic findings. The investigations demonstrated the considerable brain delivery and efficient neuronal remyelination of SLN-encapsulated MTA. Additionally, they claimed that it might safely and efficiently treat MS-like symptoms 103.

Two axo-glial-glycoprotein antigens found in the Ranvier node, anti-Contactin-2 or anti-Neurofascin, were produced and used to surface-modify PEGylated SLNs in an investigation. In contrast to the control, when the surface was altered with anti-Contactin-2 or anti-Neurofascin; accordingly, their cellular absorption was increased by 4- and 8-fold. The findings of this study will aid scientists in creating MS nanocarriers that are more effective 104.

### 6.5 Huntington Disease

Dysfunctions in mitochondria play a significant role in the development of HD. Therefore, developing methodologies to treat mitochondrial defects may offer a future therapeutic approach. To treat 3-nitro-propionic acid (3-NP)-induced HD in rats, Sandhir et al. employed curcumin-encapsulated SLNs (C-SLNs). Comparing C-SLN-treated rats to 3-NP-treated rats, the C-SLN-treated rats' neuromotor coordination significantly improved, and other parameters like mitochondrial swelling, lipid peroxidation, protein carbonyls, and reactive oxygen species were significantly decreased. As a result, this study's findings imply that administering C-SLNs may be a practical therapeutic approach to treating HD 105.

Bhatt et al. successfully used heat homogenization to create SLNs that included Rosmarinic acid (RA). Treatment with RA-loaded SLNs can considerably lessen 3NP-induced deficiencies in body weight, beam walk, locomotor, motor coordination, and striatal oxidative stress. According to the results of this investigation, nasal injection of RA-loaded SLNs may be a potential strategy for managing HD 106.

The goal of Ramachandran & and Thangarajan was to compare thymoquinone solution (TQ-S) with solid lipid nanoparticles encapsulated with thymoquinone (TQ-SLNs) against 3-NP-induced behavioral despair, oxidative damage, and striatal pathology. The investigation's findings suggest that a moderate amount of TQ-SLNs dose is more than enough to achieve the effect of TQ-S to minimize behavioral, biochemical, and histological abnormalities in HD animals exposed to 3-NP 107. Verma et al. suggested that Engeletin-nanostructured lipid nanocarriers can enhance transport and boost bioavailability when used to treat HD, which may result in treating or preventing this devastating condition 108.

## 7. Future Perspectives and Concluding Remarks

Research is an ongoing process, and research on neurodegenerative disorders is the most prominent topic for several sectors, like pathology, pharmacology, medicinal chemistry, drug discovery, and drug delivery sciences. Many people suffer from various neurodegenerative disorders, and scientists worldwide are trying to find effective therapeutics for those diseases. Since these diseases usually originate in the brain, we need to deliver drugs to the specific target site of the brain to treat the conditions, and most of the promising drug candidates fail to pass the blood-brain barriers. That is why scientists' focus is not only on discovering drugs but also on searching for an effective delivery system for drugs. In this aspect, nanoparticles shed some light on the hope of overcoming blood-brain barrier-related problems. Different research suggested that lipid nanoparticles can be a possible solution for the targeted delivery of drugs to treat neurodegenerative disorders. But, more extensive research on these fields is needed to validate their efficacy.

## Figures and Tables

**Table 1:** Lipid-based nanoformulations for the treatment of neurodegenerative disorders

Drugs	Formulations	Diseases	References
Piperine	Solid lipid nanoformulation of piperine	AD	109
Quercetin	Quercetin-loaded solid lipid nanoparticles	AD	86
Phosphatidic acid	Phosphatidic acid or cardiolipin-loaded liposomes and solid lipid nanoparticles	AD	110
Curcumin	Curcumin-loaded solid lipid nanoparticles	CVDs	111
3-n-Butylphthalide	3-n-Butylphthalide (dl-NBP) in PEGylated-lipid nanoparticles (PLNs) in conjugation with Fas ligand antibody	CVDs	112
Edaravone	Edaravone-loaded lipid-based nanosystem (LNS)	CVDs	113
Rivastigmine	Solid lipid nanoparticle formulation of rivastigmine	AD	114
Tarenflurbil	The intranasal solid lipid nanoparticle of tarenflurbil	AD	115
Fibroblast growth factor (bFGF)	Gelatin-based nanostructured lipid carriers encapsulating fibroblast growth factor (bFGF)	PD	116
Apomorphine	Apomorphine-containing solid lipid nanoparticles (SLNs) with glyceryl monostearate (GMS) and polyethylene glycol monostearate (PMS) incorporated emulsifiers.	PD	117
Curcumin and piperine	Co-loading of curcumin and piperine in lipid-based nanoparticles	PD	118
Dimethyl fumarate (DMF)	lipid-based nanoparticles loaded with dimethyl fumarate (DMF)	MS	102
Phosphatidylserine and phosphatidic acid	Liposomes, prepared by phosphatidylserine and phosphatidic acid	MS	119
Pomegranate seed oil (PSO)	Nanoemulsion formulation of pomegranate seed oil	MS	120
Engeletin	Engeletin-nanostructured lipid nanocarriers	HD	108
CAG-siRNA	Lipid nanoparticles delivered cytosine-adenine-guanine (CAG)-trinucleotide-siRNA	HD	121

Here, AD: Alzheimer's disease; CVDs: Cerebrovascular diseases; PD: Parkinson's disease; MS: Multiple sclerosis; HD: Huntington's disease
